# Supplementary figures and images for: Positive selection within the genomes of SARS-CoV-2 and other Coronaviruses independent of impact on protein function
Source: PeerJ. 2020 Oct 16;8:e10234. doi: 10.7717/peerj.10234 (PMC7571416; doi:10.7717/peerj.10234)

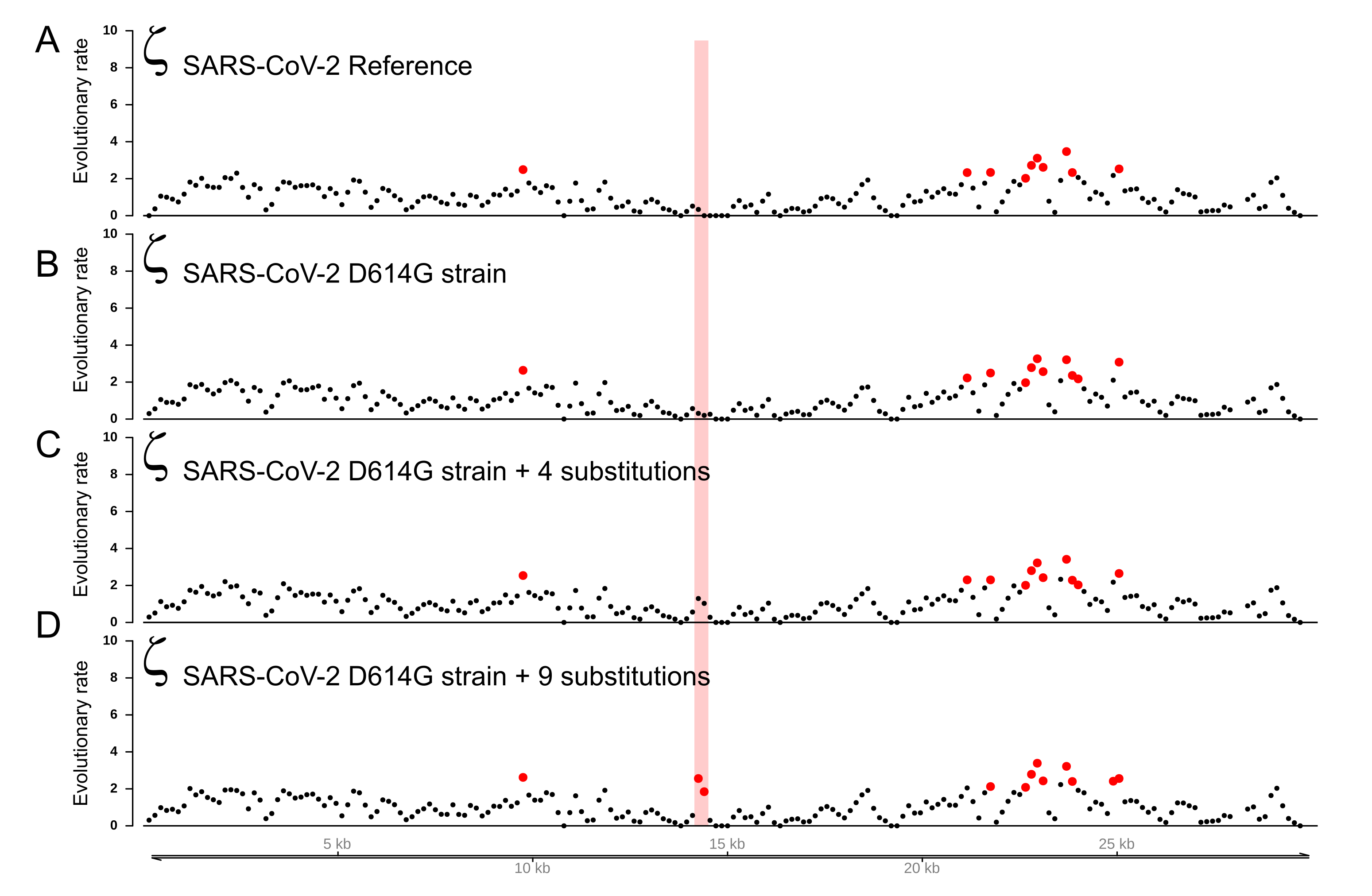

Supplement: Supplemental Information 3 — (A) Distribution of selection along the genome of SARS-CoV-2 reference genome. (B) Here we used a strain of SARS-CoV-2 that is currently leading the pandemic sampling (we called D614G strain based on its mutation in the Spike protein). (C) Four artificial mutations added in the vicinity of the mutation at 14,408 bp of D614G strain. (D) Nine artificial mutations added in the vicinity of the mutation at 14,408 bp of D614G strain. [file peerj-08-10234-s003.png]

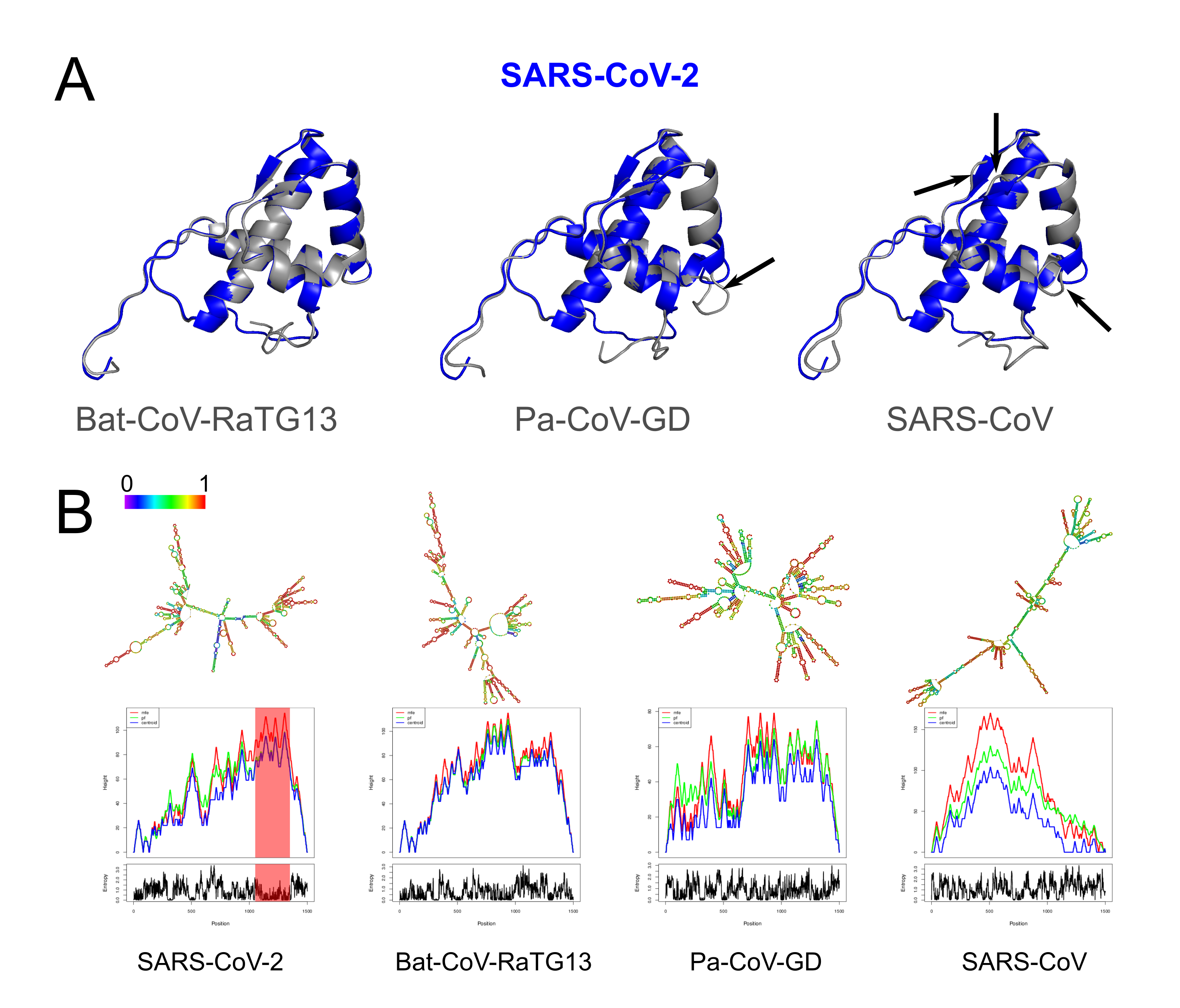

Supplement: Supplemental Information 4 — (A) Tertiary predictions of Nsp4 of SARS-CoV-2 (red) overlapping with three other species (gray). We observe structural differences in the overlap of Nsp4 between SARS-CoV-2 and Pa-CoV-GD, and SARS-CoV-2 and SARS-CoV (black arrows). (B) Thermodynamic ensemble predictions and MFE mountain plots for Nsp16 in SARS-CoV-2, Bat-CoV-RaTG13, Pan-CoV (Guangdong) and SARS-CoV at 37 °C. Regions under positive selection shaded in red. [file peerj-08-10234-s004.png]

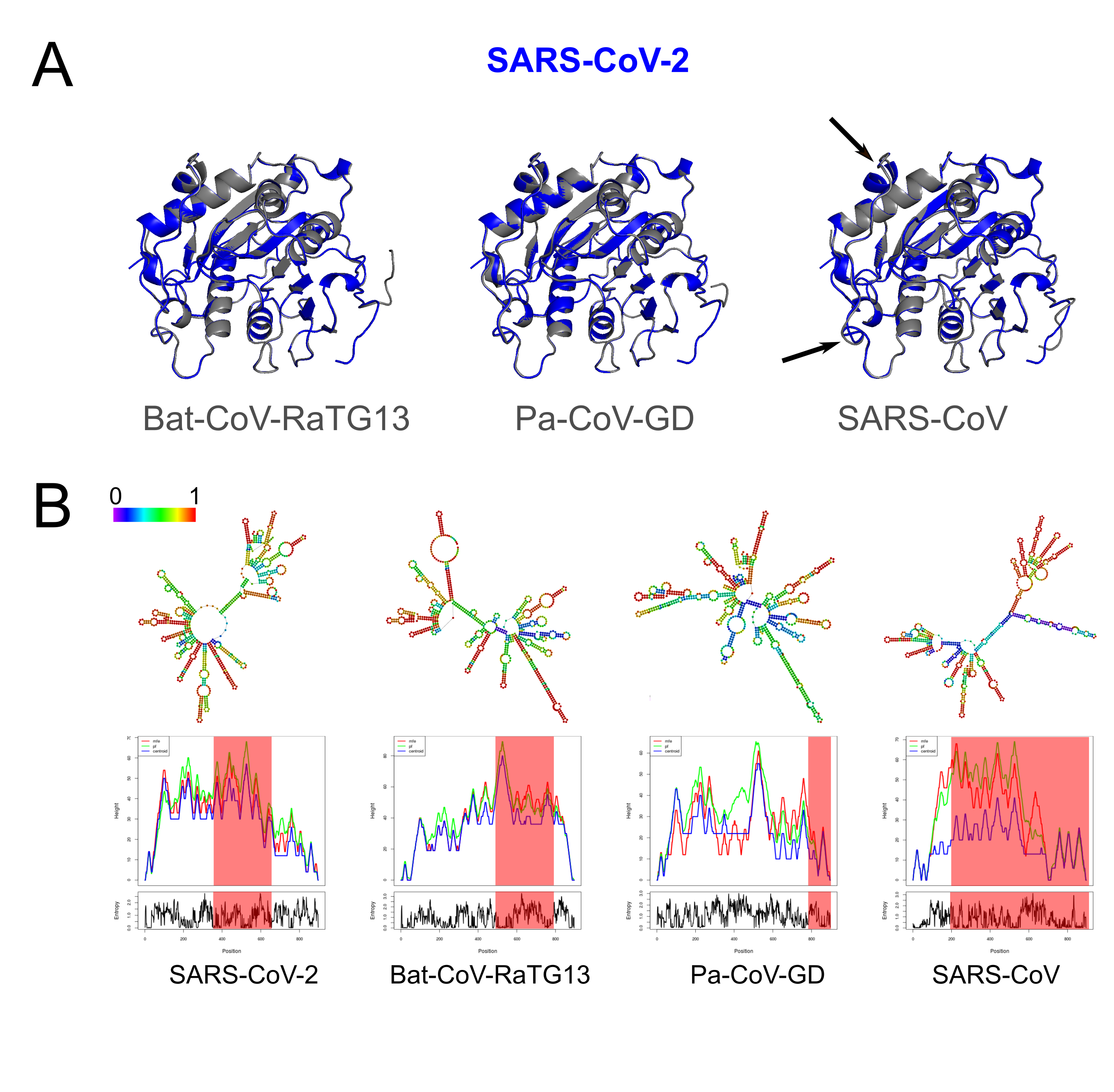

Supplement: Supplemental Information 5 — (A) Tertiary predictions of Nsp16 of SARS-CoV-2 (red) overlapping with three other species (gray). We observe structural differences in the overlap of Nsp16 between SARS-CoV-2 and SARS-CoV (black arrows). (B) Thermodynamic ensemble predictions and MFE mountain plots for Nsp16 in SARS-CoV-2, Bat-CoV-RaTG13, Pan-CoV (Guangdong) and SARS-CoV at 37 °C. Regions under positive selection shaded in red. [file peerj-08-10234-s005.png]

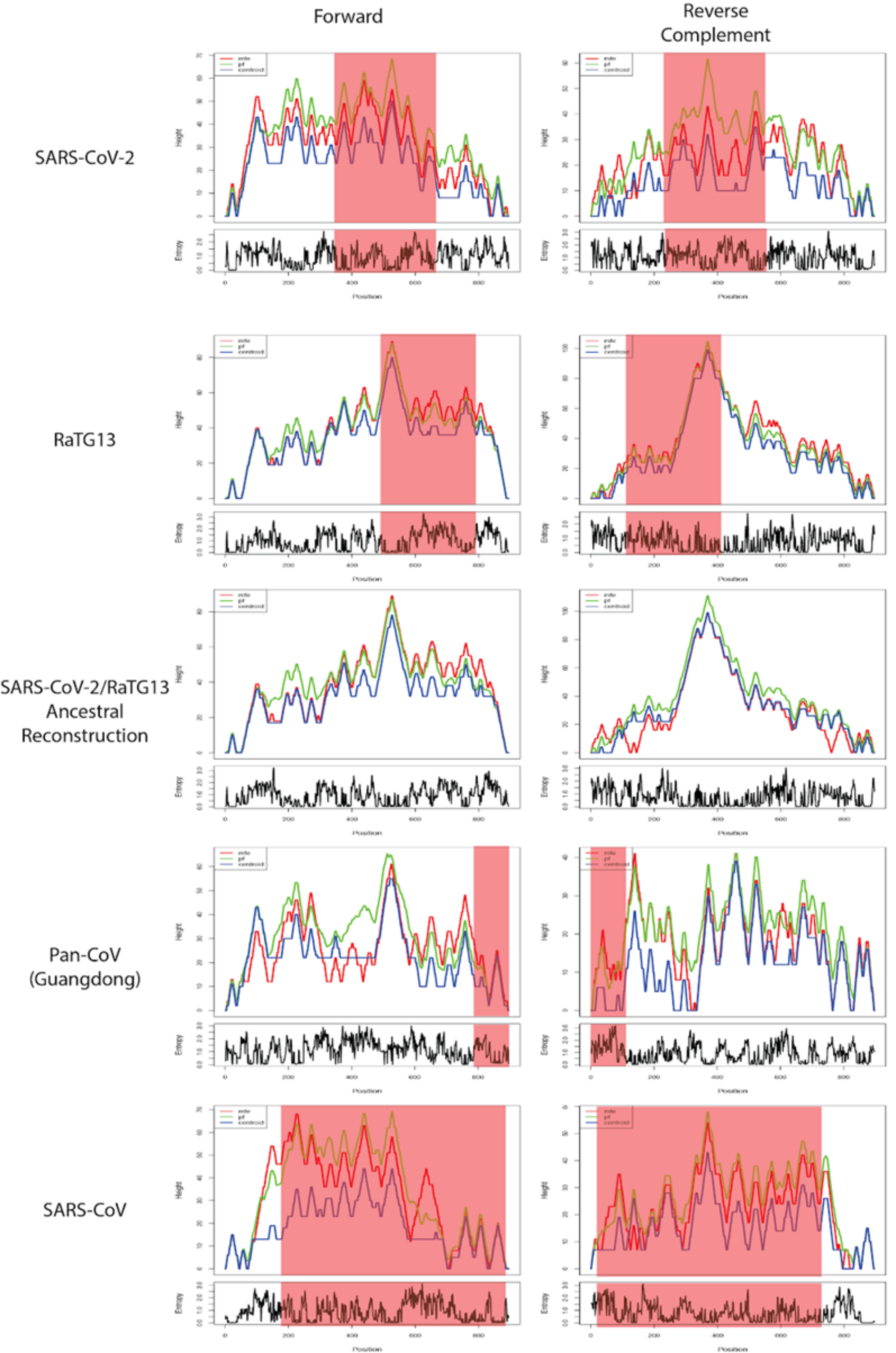

Supplement: Supplemental Information 6 — MFE mountain plots for the forward and reverse strands of Nsp16 in SARS-CoV-2, Bat-CoV-RaTG13, the reconstructed ancestor of SARS-CoV-2 and RaTG13, Pan-CoV-GD and SARS-CoV at 37 °C. Regions under positive selection shaded in red. [file peerj-08-10234-s006.png]

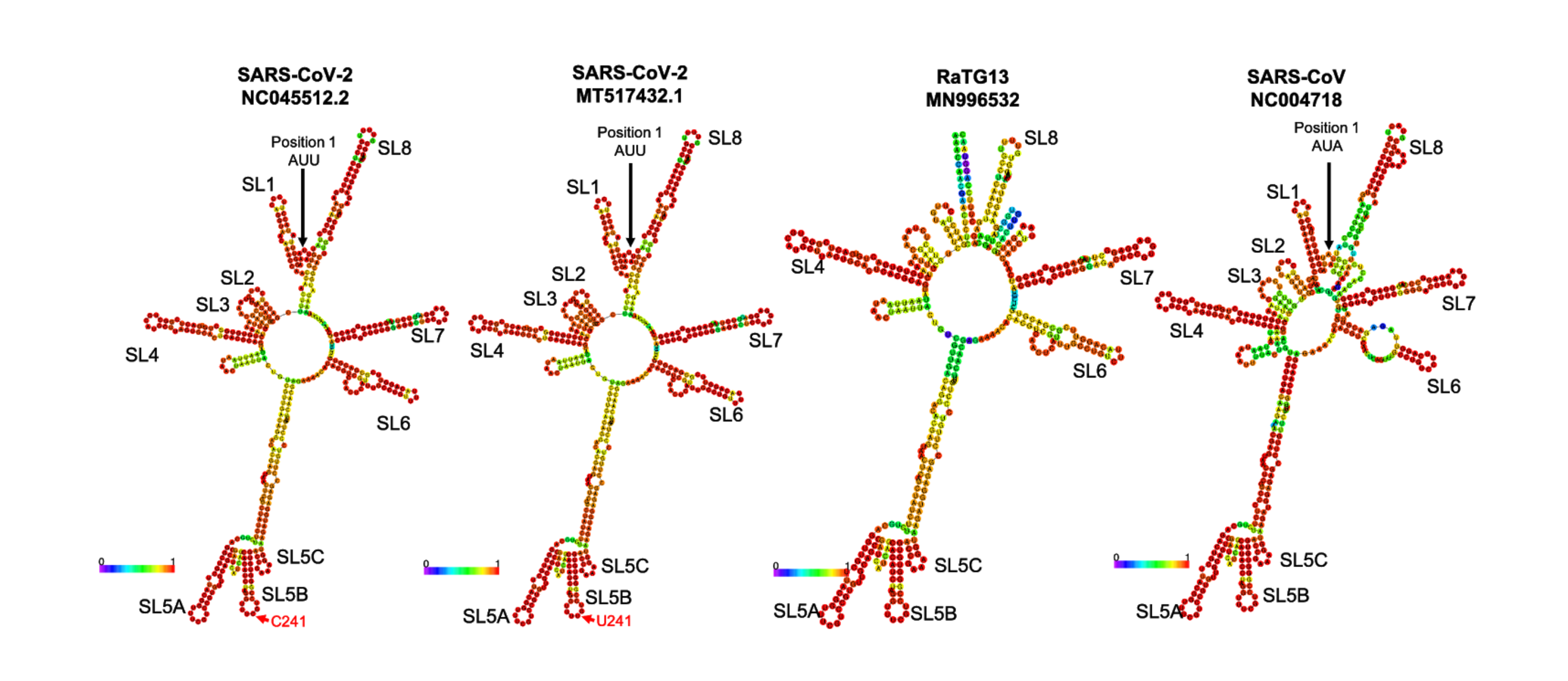

Supplement: Supplemental Information 7 — The C>U mutation at position 241 is within the SL5B stem-loop structure, indicated with a red arrow. The Pan-CoV-GD 5’ UTR sequence is missing the first 129 bp relative to the Wuhan SARS-CoV-2 reference sequence and so is not included here. [file peerj-08-10234-s007.png]

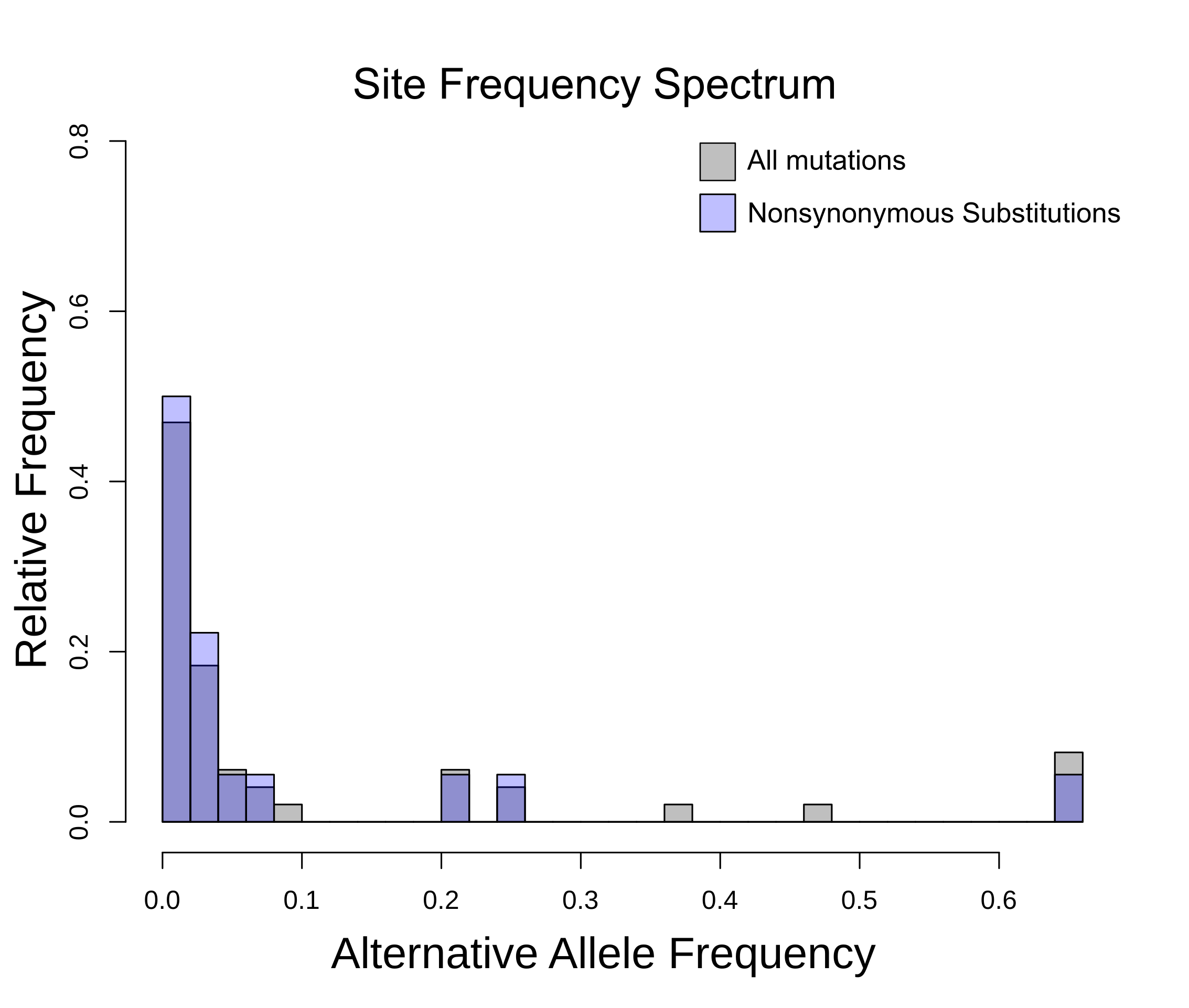

Supplement: Supplemental Information 8 — The alternative allele frequency in 5,000 SARS-CoV-2 genomes is depicted on the x axis, and the y axis shows the relative frequency of each mutation. The alternative alleles were inferred by referencing SARS-CoV-2 genomes to the NC_045512 reference genome. [file peerj-08-10234-s008.png]
