# Supplementary material for: Positive selection within the genomes of SARS-CoV-2 and other Coronaviruses independent of impact on protein function
Source: PeerJ. 2020 Oct 16;8:e10234. doi: 10.7717/peerj.10234 (PMC7571416; doi:10.7717/peerj.10234)
Supplement: Supplemental Information 9 — Pairwise comparisons of structure similarity by FATCAT shows all were significantly similar. [file peerj-08-10234-s009.docx]

**Supplementary Table**

|  | **SARS-CoV-2 vs RATG13** | | | **SARS_CoV-2 vs SARS-CoV** | | | **RATG13 vs SARS-CoV** | | | **Ancestral Seq vs SARS-CoV-2** | | | **Ancestral Seq vs RATG13** | | | **Ancestral Seq vs SARS-CoV** | | |
| --- | --- | --- | --- | --- | --- | --- | --- | --- | --- | --- | --- | --- | --- | --- | --- | --- | --- | --- |
|  | **RMSD (Å)** | **P-value** | **No. Twists** | **RMSD (Å)** | **P-value** | **No. Twists** | **RMSD (Å)** | **P-value** | **No. Twists** | **RMSD (Å)** | **P-value** | **No. Twists** | **RMSD (Å)** | **P-value** | **No. Twists** | **RMSD (Å)** | **P-value** | **No. Twists** |
| **nsp4 (whole sequence)** | **15.77** | **1.43E-02** | **5** | **6.15** | **2.52E-03** | **5** | **12.64** | **2.94E-02** | **5** | **15.84** | **2.26E-02** | **4** | **19.34** | **4.96E-02** | **5** | **12.26** | **1.87E-02** | **5** |
| **nsp4 (final 100 AA)** | **1.53** | **1.11E-16** | **0** | **2.12** | **1.61E-13** | **0** | **1.9** | **3.89E-15** | **0** | **1.82** | **4.44E-16** | **0** | **1.96** | **1.11E-15** | **0** | **2.13** | **3.65E-14** | **0** |
| **nsp5-3CL-pro** | **0.6** | **0.00E+00** | **0** | **0.22** | **0.00E+00** | **0** | **0.29** | **0.00E+00** | **0** | **0.33** | **0.00E+00** | **0** | **0.34** | **0.00E+00** | **0** | **0.3** | **0.00E+00** | **0** |
| **nsp16** | **0.17** | **0.00E+00** | **0** | **0.31** | **0.00E+00** | **0** | **0.33** | **0.00E+00** | **0** | **0.19** | **0.00E+00** | **0** | **0.17** | **0.00E+00** | **0** | **0.86** | **0.00E+00** | **0** |

**Supplementary Table 1.** Protein structures of Nsp4, Nsp5, and Nsp16 were predicted by PHYRE2. Pairwise comparisons of structure similarity by FATCAT shows all were significantly similar.
